# Nature-Based Environment as a Workplace of Forest Therapy Specialist in Healthcare Context: Legal Perspective

**DOI:** 10.3390/healthcare14070933

**Published:** 2026-04-03

**Authors:** Gintarė Tamašauskaitė-Janickė, Daiva Petruševičienė

**Affiliations:** Department of Rehabilitation, Faculty of Nursing, Lithuanian University of Health Sciences, LT-50161 Kaunas, Lithuania; daiva.petruseviciene@lsmu.lt

**Keywords:** forest therapy, forest bathing, workplace, nature-based environment, healthcare, law, legal liability, forest therapy specialist, forests management, land use

## Abstract

**Highlights:**

**What are the main findings?**

- Forest therapy legal frameworks at the international, EU, and national levels are fragmented, particularly regarding their integration into healthcare systems and the regulation of nature-based environments as professional workplaces.
- Interdisciplinary coherence between health and forest policy is a key factor influencing the practical implementation of forest therapy as a healthcare service and the management of emerging legal relationships.

**What are the implications of the main findings?**

- The absence of clear and integrated legal regulation increases legal uncertainty related to employment conditions, professional liability, patient rights protection, and the responsible use of natural resources.
- Cross-disciplinary development of harmonized legal frameworks could support the sustainable integration of forest therapy into healthcare systems, improve employment opportunities for specialists, and strengthen the protection of the right to health across jurisdictions.

**Abstract:**

**Background/Objectives:** This study examines the legislation governing forest therapy in healthcare, centered on nature-based environments as workplaces for professional forest therapy specialists within international, EU, and national legal frameworks from a labor law perspective. **Methods:** Using systematic legal analysis, comparative document analysis, and analysis of the scientific literature, the study examines current relevant international, EU, and national (Lithuania, the Republic of Korea) regulations. **Results:** Based on a cross-sectoral legal norms analysis, the legal conception of forest therapy in healthcare systems and the general regulatory framework for the professional use of nature-based environments as workplaces were identified, along with their impact on the realization of the right to work, workplace requirements, and the provision of forest therapy services. Regulatory mechanisms and conditions governing the use of nature-based environments for forest therapy purposes, under schemes administered by public and private bodies, were identified and analyzed. The interaction between nature-based workplace factors and legal liability arising from professional, contractual, and service-based relationships was also defined and clarified. **Conclusions:** Fragmented legal regulation of nature-based environments as workplaces for forest therapy creates legal uncertainty, limits the realization of the right to work, and increases legal risks in employment, service provision, patient protection, and resource use. Strengthened interdisciplinary integration between health and forest policy is essential to ensure service quality, accessibility, and legal certainty. Therefore, future regulation should prioritize integrated and harmonized legal frameworks that recognize forest therapy within healthcare systems, ensure fair working conditions, and establish clear rules for the professional use of nature-based environments in therapeutic practices.

## 1. Introduction

The potential role of nature-based environments in human health and well-being, as well as the health benefits of spending time in forests and other nature-based environments, have gained increasing recognition [1]. Studies indicate that forest bathing, also known as Shinrin-Yoku, contributes to better mental well-being by alleviating stress, anxiety, and depressive symptoms while enhancing overall mood [2]. Additionally, exposure to forest settings provides measurable physiological advantages, including lower blood pressure, improved cardiovascular regulation, strengthened immune responses, and reduced blood sugar levels in individuals with diabetes [3,4,5,6].

As reported by the authors [3], summarizing the results of previous studies [4,5,6,7,8,9,10,11,12,13,14,15,16,17,18,19,20,21], notable beneficial impacts on mental health, particularly among individuals with depressive tendencies, were confirmed through psychological assessments and physiological indicators, revealing improvements in mood and depressive symptoms [4,5,6], a decrease in anxiety and stress rates [7,8,9], and a reduced risk of psychosocial stress-related diseases [10,11,12]. From a physiological perspective, the forest environment showed anti-hypertensive effects [11,13,14], supported the normalization of heart rate variability [15,16,17], increased the number and activity of human natural killer cells [18,19,20], and reduced blood glucose levels in diabetic patients [21]. Due to these evidence-based benefits, forest therapy is now viewed as a valuable tool for disease prevention and has been adopted within healthcare initiatives in several countries.

Furthermore, it was found that many publications have highlighted the interdisciplinary connections of forest therapy as the method has effects on healthcare and public health [22,23,24,25], education [26,27,28], tourism [29,30,31,32], and the working environment [33,34].

Although the concept of forest therapy is not well-established in the scientific literature, forest bathing, forest therapy, and nature-based interventions are often used interchangeably. However, when standardizing practices, forest therapy is understood as “an evidence-based public health practice through guided forest therapy walks combining a specific blend of complementary physical and mental exercises in suitable forest surroundings leading to a lower heartbeat, blood pressure and stress levels while, at the same time, the immune system, breathing and the overall physical and mental fitness and agility are strengthened” [35] or as “a guided or self-led immersive experience in a forest or nature setting intended to promote physical, mental, emotional, and social well-being” [36]. Thus, from these definitions, it is understood that a nature-based setting (forest, park, or other natural environment) is an essential element required for the practical implementation of the forest therapy method.

On one hand, most of the mentioned studies are focused on the forest’s therapeutic (healing) effects to health and providing and measuring some descriptive details of the climatic conditions and forests’ or landscapes’ compositions and structures during method testing through research; on the other hand, they do not provide a detailed legal analysis of these facilities as potential forest therapy specialist workplaces. Certain authors recognized forest therapy in the forest policy context and discussed the need to develop legislation on forest welfare services [37]. Nevertheless, a lack of legal research on the role and integration of forest therapy within the professional healthcare system remains.

Thus, forest therapy has been widely discussed in existing scholarship for its potential benefits to human well-being; however, the existing literature has largely focused on its positive effects, with limited attention to potential risks and challenges. This study adopts a more balanced perspective by examining the legal and practical issues involved in integrating forest therapy into healthcare systems. Drawing on comparative analysis and case studies, it explores how this emerging practice, as implemented across different jurisdictions, may affect the realization of human rights related to healthcare. Attention is given to legal risks, regulatory gaps, and key areas of concern, such as patient rights, professional standards, working conditions, forest use, and legal liability.

The integration of new nature-based methods into sensitive healthcare systems requires special attention and clear legal and systematic understanding of the regulatory environment and how to harmonize legal systems and provide effective integration, balanced among other social values such as the right to work, protection of property rights, ensuring the accessibility of services, providing appropriate working conditions for forest therapy specialists, protection of the legitimate interests of patients, addressing issues related to forests, land or natural resource use, and reviewing forest and land management practices.

This study aims to examine the key elements relevant to implementing the right to work and nature-based environments as workplaces for professional forest therapy specialists within the healthcare context, primarily from the perspective of labor law principles.

This article is expected to contribute valuable insights to interdisciplinary legal research in the field of social sciences through the results of this pilot research project and provide new original knowledge by placing existing and future research within an appropriate legal context on forest therapy, while supporting the integration of this method into public health and healthcare policy agendas, legal systems, and practices.

## 2. Materials and Methods

This study is based on a systematic legal and literature review, combined with comparative document analysis, to examine the legal and professional framework for nature-based environments as workplaces for forest therapy specialists in healthcare. The analysis draws on topic-related scientific doctrine, as well as international, EU, and national legal frameworks, focusing on selected jurisdictions: Lithuania—recognized as the first EU country to legally acknowledge forest therapy as a complementary and alternative healthcare service through a dedicated law—and the Republic of Korea, which has established a foundational legal framework for forest therapy through forest policy and management schemes. Precise final definitions of forest therapy, forest bathing, and nature-based environments as professional workplaces were not predetermined. For legal analysis, the study primarily considers definitions established in legally binding instruments, while also considering the diverse ways these concepts are used in the scientific literature.

Source identification: Scientific publications were collected from Academic Search Ultimate (EBSCO), ScienceDirect, MDPI, and other interdisciplinary databases, focusing on sources indexed in Scopus or Web of Science and published between 2005 and 2025. Legal documents were obtained from EUR-Lex (EU context), the E-tar system (Lithuania), and *KLRI* (Republic of Korea), including relevant acts adopted up to 31 December 2025. Case-law searches were performed in the Case-Law Database of the Court of Justice of the European Union (2005–2025) using the term “forest therapy”, and in the Lithuanian LITEKO system (2020–2025) using the search string “miško terapij*”. Publicly available information from official websites of forest therapy-related service providers, responsible public bodies, and other stakeholders was also included, focusing on materials that were current and relevant during 2025 and Q1 2026.

Selection criteria and principles applied: The scientific literature included studies on forest therapy, forest bathing, nature-based interventions, related legal frameworks, and health issues; excluded sources were not peer-reviewed or unrelated to health or legal contexts. Legal and regulatory documents included various international, EU, and national regulatory instruments: conventions, directives, laws, regulations, policies, and non-binding guidelines directly addressing forest therapy, workplace conditions, and healthcare practice; drafts and documents not publicly accessible were excluded. The initial screening did not yield any court decisions relevant for further analysis.

Analytical framework: Comparative analysis was operationalized across the selected jurisdictions in line with the overall research design, focusing on the positioning of forest therapy within different policy fields and legal frameworks (healthcare systems versus forest policy). From a legal perspective, the analysis further explored nature-based environments as potential workplaces for professional forest therapy practitioners in healthcare. This allowed systematic identification of similarities, differences, and legal gaps between Lithuania and the Republic of Korea. These jurisdictions were selected based on the research project’s design, scope, and limitations, allowing for more in-depth analysis through research visits.

## 3. General Legal Framework of Nature-Based Environment as a Workplace of Forest Therapy Specialist

### 3.1. Legal Understanding of Nature-Based Environment for Professional Forest Therapy Practice

In principle, labor law recognizes a workplace as a place where an employee performs work agreed upon in an employment contract or performs public administration functions [38]. Working conditions encompass the work environment, meaning the space surrounding the workplace where physical, chemical, biological, and other hazardous or harmful risk factors may be present, as well as work equipment, the nature of work, working and rest time, and other factors affecting an employee’s well-being, performance, safety, and health [39]. Employee safety and health refer to all preventive measures intended to preserve employees’ working capacity, health, and lives in the workplace, implemented or planned at all stages of a company’s activities to protect employees from occupational risks or to minimize them as much as possible [40].

According to some authors, forest composition significantly influences recreational outcomes in varied ways across and within forest types and incorporating this perspective can enhance recreation planning and climate change adaptation [41]. The researchers highlight forest value orientations and the importance of forest recreation services, noting that spiritual values relate to the calming, aesthetic, and health-promoting benefits of forests, while cultural values emphasize the conservation of indigenous forest heritage [42]. The other authors outline a preference for mixed forests for forest therapy purposes [43]. Scientific publications indicate that the development of multifunctional urban green spaces can support forest therapy for residents and city amenities, while biological volatile organic compounds provide health benefits by reducing pollutants, relieving stress, and supporting therapeutic and commercial uses [44].

Better understanding of practical expectations from nature-based environments as potential workplaces of forest therapy specialists provides a valuable research study that through an iterative, guided approach identifies three site-related criteria—landscape character and quality, tranquility, and accessibility—and two trail-related criteria—design and construction, and key features and qualities, each further detailed by sub-criteria specifying conditions and considerations for effective forest therapy trail development [45].

At the same time, the review identified five problematic aspects of forest therapy recreation, namely ecosystem impacts, visitor safety and health, recreation outcomes, service and operations, and tourism planning, and concluded that forest therapy activities can burden ecosystems, pose safety risks, produce uneven recreational effects, generate management conflicts, and face planning challenges such as overuse and inadequate facilities, highlighting the need for future low-impact development strategies [46]. Other authors recommend paying attention to the safety and potential efficacy of complementary and alternative medicine therapies when administered by users themselves or by unlicensed practitioners [47]. Thus, these risks are related to legal approaches in different ways.

Professional healthcare provider services, by their nature, are a state-regulated professional activity aimed at the timely diagnosis and prevention of health disorders, as well as at supporting the recovery and strengthening of individual health, and are a sensitive part of the right to health legal framework.

The right to health is recognized as a fundamental human right in international law. It is explicitly affirmed in the Preamble of the Constitution of the World Health Organization (WHO), which states that the enjoyment of the highest attainable standard of health is one of the fundamental rights of every human being [48]. Furthermore, Article 25 of the Universal Declaration of Human Rights [49] establishes the right to an adequate standard of living necessary for health and well-being, including medical care. The right to health is most clearly articulated in Article 12 of the International Covenant on Economic, Social and Cultural Rights [50], which recognizes the right of everyone to the highest attainable standard of physical and mental health. At the regional level, the European Social Charter [51] guarantees the right to health protection under Article 11. Article 35 of the EU Charter of Fundamental Rights guarantees that everyone has the right to access preventive healthcare and to receive medical treatment in accordance with national laws, while ensuring that a high level of human health protection is taken into account in the development and implementation of all EU policies and activities [52]. The implementation of the right to health is ensured through national legislation, depending on the jurisdiction.

From a labor law perspective, international, European, and national labor law instruments consistently recognize workers’ right to safe, healthy, and dignified working conditions, imposing obligations on both States and employers to ensure appropriate workplace environments and effective occupational health protection.

According to Article 23 of the Universal Declaration of Human Rights [53], the right to just and favorable conditions of work includes protection against unemployment and respect for human dignity in the workplace. In accordance with Article 7 of the International Covenant on Economic, Social and Cultural Rights [54], the right of everyone to just and favorable conditions of work, ensuring safe and healthy working conditions, fair wages, rest, leisure, and reasonable limitation of working hours, was established. Article 31 of the EU Charter of Fundamental Rights [55] establishes that every worker is entitled to working conditions that respect their health, safety, and dignity, including limitations on maximum working hours, entitlement to daily and weekly rest periods, and an annual period of paid leave. Article 3 of the European Social Charter [56] outlines the right to safe and healthy working conditions, obliging States to formulate, implement, and periodically review national policies on occupational safety and health. In accordance with Article 16 of the International Labour Organization (ILO) Convention No. 155 on Occupational Safety and Health [57], States are required to ensure, through national policy and legislation, that working environments are safe and without health risks. ILO Convention No. 161 on Occupational Health Services [58] establishes workers’ right to occupational health services that prevent work-related injuries and diseases and promote both physical and mental well-being. Council Directive 89/391/EEC (Framework Directive on Safety and Health at Work) [59] sets out general principles for the protection of workers’ safety and health in the European Union, including the employer’s duty to ensure safe working conditions and appropriate work environments that must be implemented in national law.

The research findings revealed that the relationship between forest therapy practice or service and the nature-based environment as a workplace depends on the way the forest therapy method is embedded in the legal system.

#### 3.1.1. Republic of Korea

The Republic of Korea was the first global country to develop a comprehensive policy framework for forest welfare services, encompassing legal regulation, institutional governance, infrastructure, and specialized professional expertise [37].

The three special laws, the Forestry Culture and Recreation Act (with amendments in 2023) [60], the Forest Education Promotion Act (with amendments in 2021) [61], and the Forest Welfare Promotion Act (with amendments in 2022) [62], directly regulate issues related to forest welfare services as an area, by its nature, closely associated with forest therapy in healthcare. In accordance with Article 2 para. 1 of the Forest Welfare Promotion Act, “forest welfare” means financial, social, and emotional assistance designed to enhance the people’s well-being by providing them with forest-based welfare services. “Forest welfare services” refer to services provided in forests, such as forestry culture and recreation, forest education, and forest therapy (Article 2 para. 2 of the Forest Welfare Promotion Act).

According to Article 2 para. 4 of the Forest Education Promotion Act, “forest healing (therapy)” refers to activities that enhance immunity and overall well-being by engaging with various forest elements, such as natural aromas and landscapes, and as a place, “a healing forest” is a designated forest area, including its land and facilities, specifically designed to support healing activities (Article 2 para. 5 of the Forest Education Promotion Act). Thus, in South Korea, forest therapy is not de jure part of the legal health system, but as a method is oriented to health-related outcomes integrally with forest policy and the forest land using the regulatory framework [63] and mandatory provisions on forest therapy instructors’ professional accreditation, working conditions, or necessary infrastructure. Article 22-2 para. 5 of the Forestry Culture and Recreation outlines “forest paths for relaxation and healing” as paths for activities to promote health, such as relaxation and healing, or for recreation in forests; in accordance with Article 22-2 the Minister of the Korea Forest Service is authorized to develop and carry out a nationwide basic plan for the establishment and management of forest paths, categorized by forest path type, every five years, aiming to encourage activities such as hiking, trekking, forest-based leisure sports, recreation, and healing (forest therapy). As required by law, the basic plan for forest paths shall outline the overall goals and policy directions, assess current demand and conditions as well as prospects, define systems for promoting forest path development and management infrastructure, establish and operate forest path information networks, and address other key policy matters related to forest paths.

**Figure 1:**
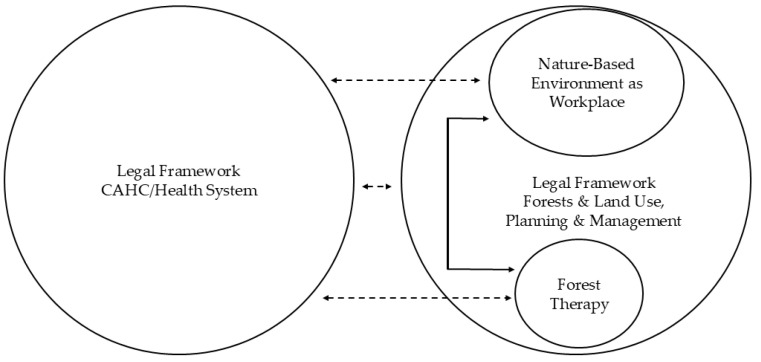
Fragmented links between the legal frameworks of health systems, forest therapy in healthcare, and the use, planning, and management of nature-based environments as workplaces for forest therapy. Double-headed arrows indicate interactions between components: a solid black double-headed arrow represents a direct connection between forest therapy and nature-based environment as a professional workplace of forest therapy specialists, regulated by forests and land use, planning, and management law; dashed black double-headed arrows indicate weakly defined connections between CAHC/health system and forests, land use, planning, and management legal frameworks; legal CAHC/health system framework and forest therapy realization; legal CAHC/health system framework and nature-based environment as a workplace for forest therapy. Source: Compiled by the authors.

Figure 1 highlights potentially limited connections between the (CA)HC and the health system, the implementation of forest therapy as part of healthcare, the nature-based workplace, and the integration of forests with land use planning and management. These gaps stem from a lack of interdisciplinary synergy in the development of the legal frameworks.

Thus, the national legal framework in the Republic of Korea reflects a general understanding of the professional nature-based environments associated with forest therapy —namely, relaxation and healing—within the scope of forest policy. However, these functions are not formally integrated into the national health system; instead, they are addressed through legal provisions governing the designation, preparation, and adaptation of areas within land and forest management. Such a legal framework ensures the conditions and guarantees necessary for the practical implementation of forest therapy by defining the forest as a nature-based environment and professional workplace subject to purpose-oriented use.

#### 3.1.2. Lithuania

In Lithuania, forest therapy is a part of the CAHC service system. The special law on CAHC of the Republic of Lithuania (CAHC Law) came into force on 1 July 2021 [64] and regulates the conditions for engaging in CAHC in the Republic of Lithuania.

Thus, in Lithuania, under Article 2 para. 2 of the CAHC Law, the CAHC is recognized as state-licensed healthcare available to citizens, residents, and legal entities of Lithuania, EU/EEA Member States, or other authorized persons, which “include health recreation, natural and traditional medicine and are carried out by means of research-based medicine, biological, psychological and social tools and/or empirical knowledge”.

In addition, the Lithuanian Medical Standard MN 189:2025 “CAHC Forest Therapy Specialist” was approved by the Ministry of Health in May 2025 (CAHC Forest Therapy Specialist Medical Standard) [65]. This medical standard is binding and applies to all forest therapy specialists working in Lithuania, their employers, and institutions responsible for their training, professional development, and supervision. The normative act provides general provisions on forest therapy services, fields of activity, rights, duties, and competencies of forest therapy specialists (the issue of professional qualifications of forest therapy specialists is recognized as important in healthcare; however, due to the scope of this study, it is not examined in detail and requires separate, dedicated research). Moreover, the CAHC Forest Therapy Specialist Medical Standard provides legal terms and definitions of forest therapy and forest therapy service, especially in the healthcare context:

- “Forest therapy”—an effect experienced by a patient in a forest or park through the scents, sounds, sights, air, and other elements of living and non-living nature, aimed at alleviating ailments caused by diseases and health disorders, and for disease prevention and health promotion (CAHC Forest Therapy Specialist Medical Standard, para. 3.1.).
- “CAHC forest therapy service”—a service provided in a CAHC institution holding a license for forest therapy within the CAHC group of biological effect services in the field of traditional medicine, by a licensed CAHC forest therapy specialist (CAHC Forest Therapy Specialist Medical Standard, para. 3.2.).

**Figure 2:**
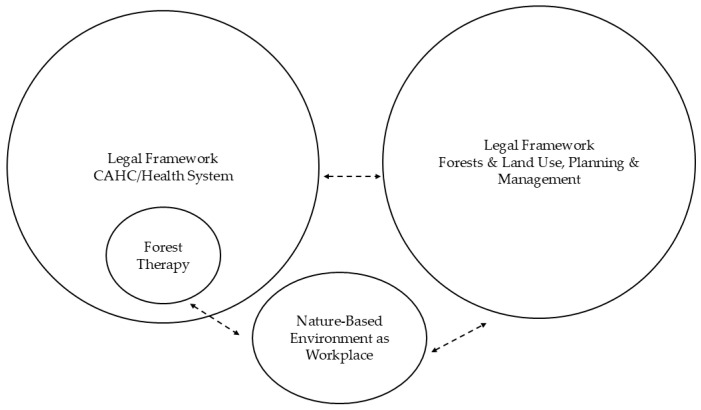
Fragmented links between the legal frameworks of health systems, forest therapy in healthcare, and the use, planning, and management of nature-based environments as workplaces for forest therapy. Double-headed arrows indicate interactions between components. Dashed black double-headed arrows represent weakly defined connections between forest therapy (regulated by health law) and nature-based environment as a professional workplace of forest therapy specialists; CAHC/health system and forests, land use, planning and management legal frameworks; nature-based environment as a professional workplace of forest therapy specialists, forest therapy realization and forests, land use, planning and management legal framework. Source: Compiled by the authors.

Figure 2 presents potentially limited connections between the (CA)HC and the health system, the implementation of forest therapy as part of healthcare, nature-based workplaces, and the integration of forests into land use planning and management. These gaps stem from a lack of interdisciplinary synergy in the development of the legal frameworks.

Thus, based on the definitions, the provision of CAHC services is associated with a licensed legal entity, whereas forest therapy is linked to a nature-based environment, i.e., a forest or a park, characterized by the scents, sounds, sights, air, and other elements of living and non-living nature. The suitability of forests or parks for the practice of forest therapy in Lithuania is not specifically described or regulated. Still, it relates to the general conditions for access to forests under forest regulations. Such a legal framework establishes the conditions and guarantees necessary for the practical implementation of forest therapy from a health law perspective, recognizing forests and parks as nature-based environments and potential professional workplaces for purpose-oriented use. However, it does not provide clear guidance on the conditions or procedures under which forests and parks may be used for this purpose, nor does it establish guarantees for land designation or management rules, which may create challenges for the effective application of forest therapy in CAHC practice.

### 3.2. Interaction of Nature-Based Environment with Workplace Requirements Under Labour Law

Since, in Lithuania, forest therapy specialists are restricted from providing services independently as a natural person, under Lithuanian labour law forest therapy specialists should therefore be employed by an institution providing CAHC forest therapy services, which must be licensed in accordance with the procedure established by law as a legal entity and ensure that forest therapy practice within the institution is carried out exclusively by licensed forest therapy specialists.

Following this conceptual approach, the relationship between CAHC institutions and forest therapy specialists should be governed by employment relations, including the application of norms concerning the provision of necessary work equipment, occupational safety and health at the workplace, and related matters.

By contrast, the results of the analysis demonstrated that legal norms establishing the regulatory framework for forest therapy in CAHC do not define a forest or park as a workplace, nor do they set requirements for such a workplace or establish special conditions for access to forests or green spaces in comparison to the common understanding of a forest or park. Nevertheless, in theory, CAHC forest therapy institutions remain under an obligation to ensure the safety and health of the professional forest therapy specialists they employ.

In other cases, where national law does not recognize the activity of a forest therapy specialist as a profession, or where forest therapy is not regulated as a component of healthcare, legal risks may arise both regarding the protection of forest therapy clients’ (patients’) rights and the assurance of healthy and safe working conditions for the forest therapy specialists themselves.

### 3.3. Nature-Based Environment Impact on Legal Liability

Nature-based environments as workplaces for forest therapy realization can create both opportunities and legal risks. Employers and employees operating in such settings must manage occupational hazards, ensure employee safety and health, protect client or patient rights, and respect the nature-based environment owned by public or private bodies, in accordance with the rules.

**Figure 3:**
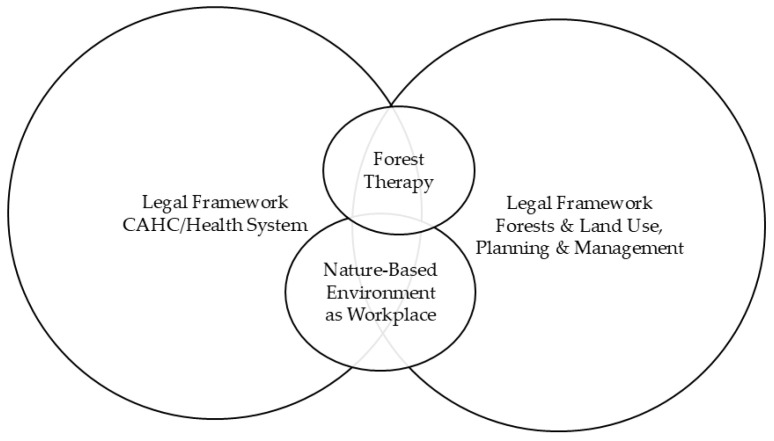
Hypothetical model of interdisciplinary legal synergy for integrating forest therapy into (complementary and alternative) healthcare or health system. Source: Compiled by the authors.

Figure 3 presents a hypothetical model in which interdisciplinary synergy between different legal frameworks enables the implementation of forest therapy within the healthcare system, ensures access to the nature-based environment required for the workplace, and clearly allocates rights and responsibilities among different stakeholders (forest therapy providers, professional forest therapy specialists, clients/patients, forests and land owners, managers, public national or local administrations and control institutions).

During the study, no court cases were found addressing issues related to forest therapy services within the healthcare sector; however, legal liability issues may arise in the future when forest therapy-related activities are not properly regulated, professional standards are unclear, or when risks to employees or clients are inadequately addressed. There is a risk that potential conflicts arise in relationships with public and private landowners and land managers regarding access to forests or other nature-based environments required for forest therapy practice, particularly when these environments are used not for personal purposes but for the provision of forest therapy when it is provided as a monetized professional service within healthcare.

For these reasons, when developing a legal framework in healthcare institutions and companies at local, national, EU, or international levels, particular attention should be paid to the clear legal conceptualized and interdisciplinary vision on the regulation of legal relationships in the following areas:

- Professional forest therapy specialist (employee) and forest therapy service provider (employer);
- Client (patient) and CAHC forest therapy specialist;
- Client (patient) and CAHC service provider;
- Forest therapy services providers and owners and managers of forests and land on the responsible use of nature-based environments as workplaces or other natural resources for forest therapy purposes in accordance with a clear legal framework;
- Forest therapy services providers and monitoring and control bodies.

In the soft law field, forest therapy-related stakeholders try to pay attention to risk management. For example, the International Society of Forest Therapy (ISFT) works [66] to develop and standardize forest therapy curricula, training, and practices while accounting for national and regional differences, and aims to make healing forests widely accessible by establishing a set of quality criteria that serve as the foundation for certifying medicinal forests. In response to the increasing diversity of forest therapy practices globally and the need to ensure their safe, ethical, and effective implementation, the Multidisciplinary Institute of Nature Therapy (MINT) at the University of British Columbia published the International Standard for Forest Therapy Practice—Framework for Health, Ecology, and Cultural Integrity (MINT Standard 2025) in 2025 [36]. The standard stresses the importance of applying international benchmarks in forest therapy, including ISO 45001 for occupational health and safety [67], ISO 26000 for social responsibility [68], the IUCN Global Standard for nature-based solutions [69], and the WHO overview of interconnectedness [70], to ensure reliable, ethical, and trusted practices for both providers and participants.

Thus, effective legal risk management, preventive measures, and clear accountability are essential to ensure liability in nature-based environments and professional forest therapy service contexts, especially in the healthcare field.

## 4. Discussion

From a legal perspective, the research results demonstrate that the legal frameworks governing forest therapy within healthcare systems, as well as related issues concerning nature-based environments as workplaces for professional forest therapy specialists, are fragmented. At the international and EU levels, there are no specific legal mechanisms regulating the realization of the right to work in the field of forest therapy as a complementary and alternative healthcare (CAHC) service or as a well-integrated part of healthcare systems.

The establishment of clear legal norms regarding the conditions and requirements for the use of nature-based environments for the provision of professional forest therapy services constitutes a fundamental basis for ensuring the sustainability and legal certainty of forest therapy activities.

A comparative analysis of the legal frameworks of Lithuania and South Korea, and their links between forest therapy and healthcare systems, revealed that interdisciplinary connections across policy areas (health policy and forest policy) have a direct impact on the practical implementation of forest therapy as a healthcare service. This is due to emerging legal risks related to developing legal relationships at multiple levels, such as the following: professional forest therapy specialist (employee) and forest therapy service provider (employer); client (patient) and CAHC forest therapy specialist; client (patient) and CAHC service provider; forest therapy service providers and owners and managers of forests and land regarding the responsible use of nature-based environments as workplaces or other natural resources for forest therapy purposes; and forest therapy service providers and monitoring and control bodies.

The lack of legal clarity affects the allocation and enforcement of legal liability, particularly in countries where no basic legal framework for forest therapy exists. On the one hand, this has a direct impact on the development of forest therapy within healthcare, a socially sensitive area due to its implications for the right to health, patient rights protection, and compensation for damage in practice. On the other hand, from a labour law perspective, the development of the forest therapy sector raises important issues concerning the working conditions and occupational safety of professional forest therapy specialists, as well as access to conditions, requirements, and opportunities for using nature-based environments as workplaces.

Additional attention should also be paid to non-mandatory certification systems (e.g., voluntary healing forest certification schemes or sector-specific standards). Forest therapy infrastructure certification by various public or private bodies represents a complex and distinct area of research, as does the recognition of the professional qualifications of forest therapy specialists. Certification schemes, particularly when applied to forests used for therapeutic purposes, may affect the integration of forest therapy into healthcare systems. As a regulatory phenomenon, such certification can influence access to forest environments, conditions of use, and compliance with environmental and land use requirements, in accordance with general forest regulations in the relevant jurisdiction. This reveals important interconnections between forest therapy as a healthcare-related method and the fragmented legal frameworks governing access to forests, land use, and forest management. These aspects indicate the need for more detailed research into the role of emerging forest certification systems within legal frameworks, their compatibility with forest management regulations, and the safeguards required to address the healthcare needs of clients or patients receiving forest therapy. Concepts aimed at defining nature-based environments through standardized instruments contribute to a better understanding of the requirements for adapting them to the professional functions of forest therapy specialists. However, in terms of legal force, these instruments remain merely recommendatory and do not always align with forest and land management mechanisms established within national jurisdictions, particularly in the context of non-timber economic development.

Legal uncertainty regarding forest therapy within healthcare systems, or a lack of clarity about the use of nature-based environments for professional forest therapy services, may, in the long term, affect employment opportunities for specialists across different countries.

While this study focuses on Lithuania and the Republic of Korea due to the research design and practical considerations, future research could expand the comparative scope to include additional jurisdictions with established experience in developing forest therapy, such as Japan, Finland, Germany, and other EU Member States, thereby enhancing the generalizability and broader applicability of the findings. Future research would benefit from broader empirical studies on the impact of legal regulation across a wider range of countries, examining additional issues directly related to the implementation of the right to work within forest therapy as a healthcare service. Such research should include legal liability, the protection and realization of clients’ (patients’) rights under different legal regimes, and an expanded understanding of nature-based environments, encompassing not only physical but also virtual environments.

## 5. Conclusions

The analysis demonstrates that forest therapy, as an emerging component of healthcare systems, currently lacks a coherent and comprehensive legal framework at both international and EU levels. The fragmentation of legal regulation, particularly concerning the use of nature-based environments as workplaces for professional forest therapy specialists, creates legal uncertainty and limits the effective realization of the right to work in this field. Clear legal norms governing the conditions, requirements, and responsibilities related to forest therapy services are therefore essential for ensuring legal certainty and sustainable sector development.

The comparative analysis of Lithuania and South Korea highlights the significant role of interdisciplinary integration between health and forest policy in the practical implementation of forest therapy as a healthcare service. The absence of coordinated legal mechanisms increases legal risks across multiple legal relationships, including employment relations, service provision, patient rights protection, and the responsible use of natural resources. This lack of clarity directly affects both the quality and accessibility of forest therapy services, as well as the legal protection of all parties involved.

Accordingly, future legal regulation should prioritize the development of integrated and harmonized frameworks that recognize forest therapy within healthcare systems, ensure safe and fair working conditions for professionals, and establish clear rules for the use of nature-based environments as workplaces. Strengthening legal coherence in this area would contribute to the long-term viability of forest therapy, enhance employment opportunities, and support the protection of the right to health and patient rights across jurisdictions.

## Data Availability

The original contributions presented in this study are included in the article. Further inquiries can be directed to the corresponding author.

## Abbreviations

The following abbreviations are used in this manuscript:

CAHC	Complementary and Alternative Healthcare
ISFT	International Society of Forest Therapy
ISO	International Organization for Standardization
IUCN	International Union for Conservation of Nature
WHO	World Health Organization
